# A study on the trajectory of change and influencing factors of care dependence in elderly patients during hospitalization after total hip arthroplasty

**DOI:** 10.1186/s12912-026-04383-8

**Published:** 2026-02-04

**Authors:** Lu Wang, Zhuoqing Wu, Hong Zhou, Yanrui Ren

**Affiliations:** 1 Department of Science and Education, The First Affiliated Hospital of Yangtze University, Jingzhou, China; 2 School of Nursing, Yangtze University, Jingzhou, China; 3 Department of Nursing, The First Affiliated Hospital of Yangtze University, Jingzhou, China

**Keywords:** Activities of daily living, Elderly, Total hip arthroplasty, Recovery of function, Risk factors, Latent class analysis

## Abstract

**Background:**

Elderly hip fracture patients (patients aged ≥ 60 years) require prolonged rehabilitation after surgery and are care-dependent. Patients’ care dependence was strongly associated with the recovery of health-related quality of life. This study aimed to explore the trajectory and influencing factors of care dependence among elderly patients 14 days after total hip arthroplasty (THA) during their hospital stay.

**Methods:**

Using the convenience sampling methodology, 210 elderly patients who underwent THA in three tertiary comprehensive hospitals in Jingzhou City and Yichang City from February to August 2023 were selected and recruited as the research subjects. The demographic and disease data questionnaire and the Orthopedic Social Support Scale were used to collect baseline information about the patients, and the Care Dependency Scale was used to follow up with the older patients at 24 h postoperatively (T1), on postoperative day 7(T2), and on postoperative day 14 (T3) for longitudinal assessment. The Latent Growth Mixture Model (LGMM) was used to describe the overall trend of care dependence, and a multivariate logistic regression model was adopted to analyze the effects of relevant factors on the trajectory of change in care dependence among elderly patients.

**Results:**

LGMM analysis revealed three distinct trajectories: a high dependency group (55.24%), a gradual decline group (37.62%), and a rapid decline group (7.14%). Compared to the high dependency group, membership in the gradual decline group was significantly associated with young-old, no complications, family members as the primary caregiver, and better social support (all *p* < 0.05). Lower education levels (both journal school and primary school or below, vs. high school or above) remained a negative predictor. For the rapid decline group, younger age and higher educational attainment (indicated by the negative effect of primary school or below education) were the significant predictors.

**Conclusions:**

Early post-THA care dependency follows heterogeneous trajectories in older patients, primarily driven by two mechanistic pathways: a compensatory pathway (where external support facilitates gradual recovery in vulnerable individuals) and an autonomous pathway (where intrinsic capacity enables rapid recovery). Early screening for age, education, and support systems allows for risk stratification and personalized interventions to improve recovery outcomes.

**Clinical trial number:**

Not applicable.

**Supplementary Information:**

The online version contains supplementary material available at 10.1186/s12912-026-04383-8.

## Introduction

According to statistics from the World Health Organization (WHO), both the number and proportion of elderly individuals are increasing globally. Currently, the population aged 60 or above has exceeded 1 billion worldwide [1]. It is projected that by 2030, one in six people globally will be aged 60 or older. By 2050, the global population aged 60 and above is expected to reach 2.1 billion, with the number of those aged 80 or older anticipated to reach 426 million. Two-thirds of the world’s population aged 60 and above will reside in low- and middle-income countries [2]. The seventh national population census conducted in 2021 revealed that the population aged 60 and above in mainland China, in which those aged 60 years or over are considered elderly, has reached 264 million, accounting for 18.7% of the total population. China has already passed through its first phase of rapid population aging and is expected to face an even more accelerated phase of population aging in the near future [3].

With the increasingly severe aging trend, the incidence of bone and joint diseases in the elderly continues to rise, and the number of patients undergoing artificial joint replacement surgery is increasing year by year, posing significant impacts on public health [4]. Hip fracture is a common injury among older adults, characterized by high incidence, high mortality, substantial risk of disability, and considerable socioeconomic healthcare costs, along with significant health losses [5]. Meanwhile, hip fractures lead to extensive loss of functional status, reduced quality of life, and long-term care needs, all of which profoundly affect patients and their families [6, 7]. Studies indicate that the global number of hip fractures is projected to increase from 1.66 million in the early 1990s to approximately 6.26 million by 2050 [8–10], with Asian patients accounting for more than 50% and elderly patients reaching about 5.4 million [8]. In China, approximately 900,000 to 1 million elderly individuals suffer hip fractures each year [11].

Total Hip Arthroplasty (THA) is an ideal surgical intervention for patients with hip fractures [12] and stands as one of the most effective orthopedic procedures [13, 14]. It offers superior postoperative function, effective pain relief, and a lower revision rate [15–17]. Multiple studies have documented significant quantitative and qualitative improvements in physical function and health-related quality of life following THA [18, 19]. Clinically, the incidence of THA has been increasing [20–22]. Studies indicate that the rates of THA are projected to increase by 43% to 70% from 2014 to 2030 [22–24]. The rates of THA surgery have risen across all age groups, with the relatively largest increase observed among patients aged ≥ 80 years [25, 26].

A U.S. study utilizing the National Inpatient Sample (NIS) database from 2005 to 2014 to examine trends in THA for femoral neck fractures found that, out of a total of 502,060 patients with femoral neck fractures, 51,568 (10.3%) underwent THA. The incidence of THA for this condition increased from 8.3% to 13.7% [4]. THA has become the preferred treatment for higher-demand elderly patients [4]. Due to the growing aging population’s higher expectations for improved mobility and quality of life, the demand for and volume of this procedure are expected to rise in the coming years [24]. Consequently, the associated healthcare burden, care demands, and societal costs are anticipated to increase [11–29].

Care dependence refers to the state in which an individual requires assistance from others to perform essential activities of daily living (ADLs), such as bathing, dressing, and mobility, due to physical or cognitive impairments. It encompasses not only functional limitations but also psychological reliance on caregivers [30]. In geriatrics, there are numerous tools designed to assess patients’ functional status, which is the first step in assessing the multidimensional care dependency of older people [31]. In this study, care dependence was operationalized and measured using the CDS scale.The scale was developed following Virginia Henderson’s theory of nursing and includes both physical and psychosocial aspects, which are used to assess care dependency [32, 33]. A score of < 68 indicates a degree of dependence, with lower scores representing higher levels of dependence.

Postoperative elderly hip fracture patients require prolonged rehabilitation exercises. Coupled with the persistent impairment of lower limb proprioception following artificial joint replacement, these patients often experience decreased muscle strength, compromised joint stability, and reduced range of motion, leading to functional decline, limited mobility, and varying degrees of self-care deficits. As a result, they tend to develop dependency on nursing staff and caregivers [7, 34–36].

Current research on nursing dependency primarily consists of cross-sectional surveys. A study conducted in Brazil involving 41 hospitalized elderly fracture patients revealed that the level of nursing dependency varies from admission to discharge [37]. Female patients exhibited higher dependency levels than males, individuals over 80 years old were more dependent than those aged 60–80, and patients with comorbidities demonstrated greater dependency.

This study dynamically observes and analyzes the developmental trajectory of care dependence during hospitalization for THA at 24 h postoperatively (T1), on postoperative day 7 (T2), and on postoperative day 14 (T3), examining heterogeneous patterns of dependency among elderly patients at different stages and identifying influencing factors. The findings aim to deepen theoretical understanding, assist healthcare providers in early identification of high-risk patients, facilitate the implementation of effective nursing interventions, and provide a basis for developing personalized care strategies. Ultimately, this approach seeks to promote rapid recovery, improve quality of life, enhance patient outcomes, and alleviate healthcare burdens.

## Methods

### Design and procedures

**Fig. 1:**
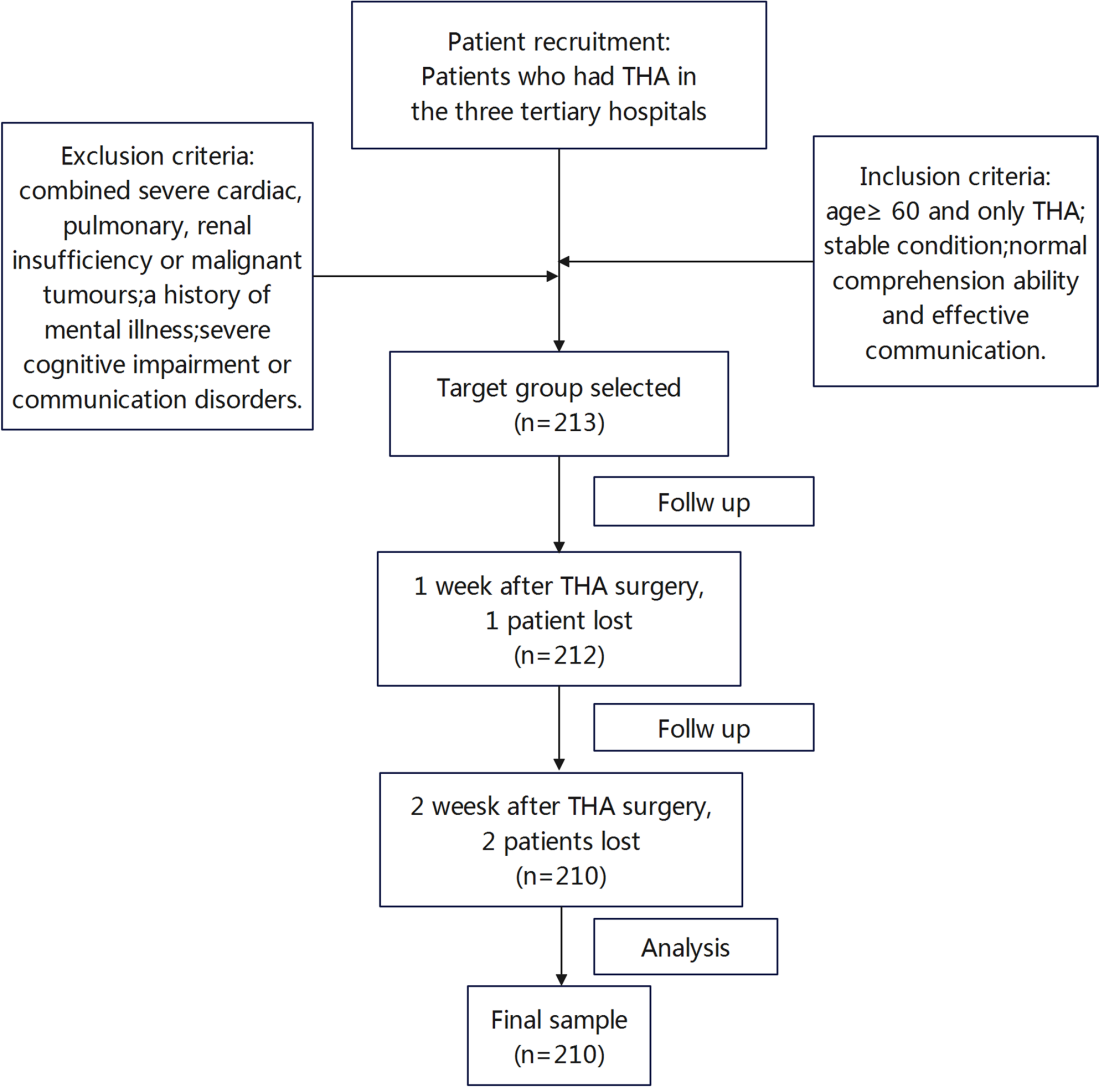
Flow diagram of the selection of sample

Using a convenience sampling method, elderly patients who underwent THA and were hospitalized in the orthopedic wards of one Grade A tertiary hospital in Jingzhou and two in Yichang between March and August 2023 were selected as study patients. The inclusion criteria were as follows: age ≥ 60 years; having undergone THA only; being in stable condition; possessing normal comprehension and effective communication abilities. Exclusion criteria included: patients with severe cardiac, pulmonary, or renal insufficiency or malignant tumors; those with a history of psychiatric disorders; those with severe cognitive impairment or communication disorders; and those unable to complete the full survey cycle.The sample size calculation was based on the rule of thumb proposed by Kendall, which recommends a minimum of 10–15 subjects per independent variable [38]. In our preliminary analysis, we planned to include approximately 15 potential predictors in the multivariate model. Thus, a minimum sample size of 150–225 patients was required. A total of 213 cases were initially included in this study. After excluding 3 cases due to failure to meet the complete survey cycle requirement, 210 cases were ultimately retained for analysis.The screening process was shown in Fig. 1.

A questionnaire survey on care dependency was administered to elderly patients at three time points: the first day (T1), the seventh day (T2), and the fourteenth day (T3) after THA surgery. Data collection was carried out through face-to-face distribution of questionnaires in the patient’s hospital room. The survey was conducted by two investigators who had undergone standardized training. They used uniform instructions to explain the purpose and significance of the survey, the method of completing the questionnaire, and important considerations for the patients. After obtaining consent, the researchers filled out the questionnaire on behalf of the patients in a question-and-answer format. This method was chosen to ensure data quality and compliance, given the advanced age and potential fatigue of the postoperative elderly patients.To ensure inter-rater reliability, both investigators received standardized training prior to the study, and their independent ratings on pilot samples achieved an intraclass correlation coefficient (ICC) greater than 0.90. When patients encountered unclear options, investigators provided explanations using standardized and objective language, avoiding any leading or suggestive expressions. If necessary, input was obtained from the patient’s assigned nurse or primary caregiver (e.g., nursing aide or family member) to complete the assessment.

Upon completion, each questionnaire was immediately checked on-site to ensure no omissions or errors before collection. Patients were promptly asked to supplement or correct any missing or incorrect responses.Questionnaires were considered invalid and excluded from the sample if any of the following applied: blank entries accounted for more than 10% of the total items and remained unresolved after explanation; responses exhibited persistent identical choices or logical inconsistencies; or the patient was unable to concentrate due to physical discomfort, resulting in termination of the survey.

### Measurements

#### General information questionnaire

After reviewing the relevant literature from domestic and international, the group designed the general demographic information questionnaire and disease information questionnaire, including age, gender, marital status, education level, monthly household income per capita, medical expense payment mode, primary caregiver, comorbidity, postoperative complications, pain score, Caprini Score for Deep Vein Thrombosis (DVT), Body Mass Index (BMI) score, and others.

### Care Dependency Scale (CDS)

The CDS was developed by Dijskra et al. [39] with a total of 15 items, including diet, elimination, body position, mobility, circadian rhythm, clothing, temperature, cleanliness, risk avoidance, communication, socialization, values and rules, daily living, recreational activities, and learning ability. The scale was based on a 5-point Likert scale, with scores ranging from 1 to 5, from “completely dependent” to “completely independent”, and total scores ranging from 15 to 75. The lower the score, the higher the degree of nursing dependence. A score of > 69 was almost independent, 60–69 was mostly independent, 45–59 was partially dependent, 25–44 was mostly dependent, and < 25 was completely dependent. The Cronbach’s alpha coefficient of the Chinese version of the CDS scale was 0.95, the inter-assessor reliability was 0.84–0.89, and the re-test reliability was 0.83–0.92, which had good reliability and validity.

### Social support scale

The scale developed by van den et al. in 2004 [40] and revised by Sheng et al. was used to assess the social support received by orthopaedic patients after hip or knee arthroplasty, which involves two dimensions and 12 entries, namely perceived social support (7 entries) and instrumental support (5 entries). A Likert 4-point scale was used, with scores ranging from 0 to 3, from “never” to “often”, and total scores ranging from 0 to 36, with higher scores indicating better social support. The Chinese version of the GO-SSS demonstrated excellent reliability and validity, with a Cronbach’s α coefficient of 0.863 and content validity index (CVI) of 0.93, supporting its robust psychometric properties for application in orthopaedic populations.

### Ethical considerations

This study was approved by the Medical Ethics Committee of the First People’s Hospital of Yichang (PJ-KY2023-02)、Yichang Central People’s Hospital(2023-017-01) and the First People’s Hospital of Jingzhou (KY202355). The ethical approval covered the enrollment of 150–225 patients. All subjects gave informed consent and participated in the study voluntarily. All methods were performed in accordance with the relevant guidelines and regulations.

### Statistical analysis

Data were entered and cross-verified by two researchers to ensure accuracy.Statistical analysis was performed using IBM SPSS Statistics for Windows, Version 26.0 (IBM Corp., Armonk, N.Y., USA) and Mplus, Version 7.0 (Muthén & Muthén, Los Angeles, CA, USA). The normality of the distribution for all continuous variables was assessed using the Shapiro-Wilk test. Continuous data with a normal distribution were presented as mean ± standard deviation and compared using one-way analysis of variance (ANOVA).Non-normally distributed data were presented as median (interquartile range) and compared using the Kruskal-Wallis H test. Categorical data were presented as numbers and percentages (n, %). Differences in categorical variables between groups were compared using the Chi-square test or Fisher’s exact test, as appropriate.

Latent Growth Mixture Modeling (LGMM) was implemented to explore the potential categories of care dependence trends and their differential characteristics at T1 ~ T3 time points. Multi-factorial analyses were performed using unordered multivariate logistic regression model. A p-value < 0.05 was considered statistically significant.

## Results

### Baseline characteristics

Among the 210 elderly patients in this study, 61.4% were male and 38.6% were female. The mean age of the older patients was 74.74 years, with a standard deviation of 9.86. Most older patients were married (71.4%), and the predominant living arrangement was cohabitation with spouses (57.6%). In terms of educational attainment, the largest proportion had primary school or lower education (35.2%). Regarding medical payment methods, urban employee basic medical insurance was the most common (46.2%). The monthly household income per capita was concentrated in the 3000–4999 RMB range (35.7%). The majority of patients were retired (60.0%). Other detailed information is presented in Table 1. During the period from T1 to T3, the total score of care dependence was (34.60 ± 8.83), (43.42 ± 13.45), and (48.41 ± 17.74), respectively.

**Table 1:** Univariate analysis of baseline characteristic in elderly THA patients

Variable	High dependency (*n* = 116)	Gradual decline group (*n* = 79)	Rapid decline group (*n* = 15)	Total	Test- statistic	*P* value
**Age(mean ± SD)**	77.28 ± 9.13	72.11 ± 9.98	68.9 ± 9.14	74.74 ± 9.86	10.06^a^	0.000
**Education level(n**,**%)**					78.677^b^	0.000
Primary school or below	62 (53.4)	11 (13.9)	1 (6.7)	74(35.2)		
Junior high school	43 (37.1)	20 (25.3)	1 (6.7)	64(30.5)		
High school or above	11 (9.5)	48 (60.8)	13 (86.6)	72(34.3)		
**monthly household income per capita(RMB)(n**,**%)**					33.577^c^	0.000
< 3000	23 (19.8)	1 (1.3)	1 (6.67)	24(11.4)		
3,000–4,999	36 (31.0)	35 (44.3)	3 (20.0)	75(35.7)		
5000–7999	46 (39.7)	21 (26.6)	5 (33.3)	72(34.3)		
≥ 8000	11 (9.5)	22 (27.8)	6 (40.0)	39(18.6)		
**Primary caregiver (n**,**%)**					39.835^b^	0.000
Spouse or child	74 (63.8)	18 (22.8)	3 (20.0)	95(45.2)		
Care attendant	16 (13.8)	30 (38.0)	8 (53.3)	54(25.7)		
None	26 (22.4)	31 (39.2)	4 (26.7)	61(29.1)		
**Comorbidity(n**,**%)**					30.665^b^	0.000
No	57 (49.1)	13 (16.5)	1 (6.7)	140(66.7)		
Yes	59 (50.9)	66 (83.5)	14 (94.3)	70(33.3)		
**Postoperative complications(n**,**%)**					30.000^b^	0.000
No	75 (64.7)	75 (94.9)	14 (94.3)	45(21.4)		
Yes	41 (35.3)	4 (5.1)	1 (6.7)	165(78.6)		
**Pain severity(n**,**%)**					24.331^c^	0.000
No pain	5 (4.3)	11 (13.9)	3 (20.0)	19(9.1)		
Mild pain	77 (66.4)	61 (77.2)	11 (73.3)	150(71.4)		
Moderate pain	34 (29.3)	7 (8.9)	1 (6.7)	41(19.5)		
**BMI categories(n**,**%)**					21.685^c^	0.001
Underweight	27 (23.3)	4 (5.1)	1 (6.7)	31(14.8)		
Normal weight	67 (57.8)	48 (60.8)	8 (53.3)	124(59.0)		
Overweight	17 (14.7)	21 (26.6)	4 (26.7)	42(20.0)		
Obese	5 (4.3)	6 (7.6)	2 (13.3)	13(6.2)		
**Social support(n**,**%)**					2.102^b^	0.000
Better	94 (81.0)	39 (49.4)	5 (33.3)	138(65.7)		
Poorer	22 (19.0)	40 (50.6)	10 (77.7)	72(34.3)		
**Time to first oral intake(mean ± SD)**	3.87 ± 3.79	2.55 ± 2.14	1.83 ± 1.14	3.23 ± 3.21	5.803^a^	0.004
**Time to first ambulation(mean ± SD)**	3.58 ± 1.43	2.97 ± 1.04	2.53 ± 0.64	3.28 ± 1.30	8.305^a^	0.000

^a^One-way ANOVA

^b^Chi-square test

^c^Fisher’s exact test

### Analysis of postoperative care dependency trajectories in elderly patients undergoing THA

**Fig. 2:**
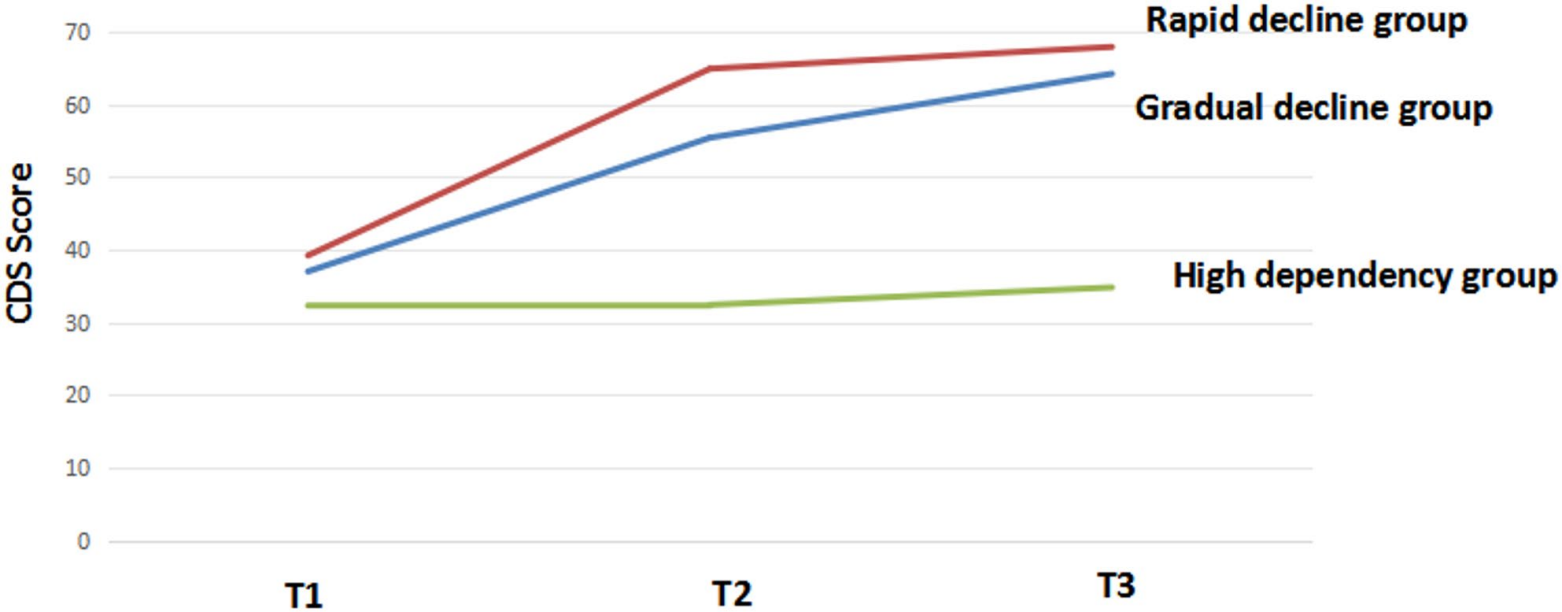
Trajectories of mean care dependency scores over time for elderly THA patients analyzed by LGMM

The model fit indices from the LGMM analysis indicated that the three-class model demonstrated superior fit compared to models with other numbers of latent classes as the number of potential categories gradually increased. The Akaike Information Criterion (AIC), Bayesian Information Criterion (BIC), and sample size-adjusted Bayesian information criterion (aBIC) values were low. The entropy value was 0.865, and both the Lo-Mendell-Rubin (LMR) and Bootstrap Likelihood Ratio Test (BLRT) reached statistical significance, indicating good classification accuracy. The parameter estimation results are presented in Table 2. Class 1 (C1) showed significantly lower care dependency scores at all three time points compared to the other two groups. The measurement scores exhibited a slight increase, indicating that the level of care dependency remained high; this group was therefore termed the “high dependency group” and comprised 55.24% of the sample. Class 2 (C2) demonstrated a steady decline in care dependency levels throughout the entire measurement period from T1 to T3, and was termed the “gradual decline group,” accounting for 37.62%. Class 3 (C3) started with a low initial level of care dependency but showed significantly higher care dependency scores during the T2-T3 period; this group was termed the “rapid decline group” and constituted 7.14% (Fig. 2).

**Table 2:** Comparison of fit indices between models

Category	AIC	BIC	aBIC	Entropy	LMR (*P*)	BLRT (*P*)	Categorical probability
1	4886.602	4903.337	4887.494	-	-	-	-
2	4804.703	4828.133	4805.953	0.85	0.000	0.000	0.55714/0.44286
3	4801.134	4831.258	4802.741	0.865	0.048	0.0253	0.37619/0.07143/0.55238
4	4803.414	4840.232	4805.378	0.806	0.5517	0.4343	0.36667/0.48095/0.07143/0.08095
5	4804.634	4808.147	4806.955	0.846	0.7928	0.2254	0.24/0.17/0.23/0.07 /0.28

### Univariate analysis of care dependency trajectories

The results of the univariate analysis showed that age(*P* = 0.000), educational level(*P* = 0.000), monthly household income per capita(*P* = 0.000), primary caregiver(*P* = 0.000), comorbidity(*P* = 0.000), postoperative complications(*P* = 0.000), pain severity(*P* = 0.000), BMI categories(*P* = 0.001), social support(*P* = 0.000), time to first oral intake(*P* = 0.004), and time to first ambulation(*P* = 0.000) had a significant influence on the distinct trajectory classes of care dependency development in elderly THA patients during the first 14 days postoperatively, with a p-value < 0.05 (Table 1).

### Multifactor analysis of the trajectory of change in care dependency

To identify independent factors associated with trajectory group membership, multivariate multinomial logistic regression analyses were performed, using the statistically significant factors from the univariate analysis as independent variables, with the high dependency group set as the reference category.

As shown in Table 3, compared to the high dependency group, several factors were independently associated with an increased likelihood of belonging to the gradual decline group. Older age (OR: 0.889 per year increase, 95% CI: 0.838–0.943, *p* = 0.000) and had postoperative complications (OR:7.141, 95% CI: 2.029–25.131, *p* = 0.002) were negative predictors, meaning that with each additional year of age or each additional postoperative complications, the odds of being in the gradual decline group decreased.In contrast, having family members as the primary caregiver (OR:0.196 compared to no caregivers, 95% CI: 0.073–0.525, *p* = 0.001) and better social support (OR = 0.245, 95% CI: 0.084–0.715, *p* = 0.01) were significant positive predictors. For education, using high school or above as the reference, both junior high school (OR:4.539, 95% CI: 1.470-14.013, *p* = 0.009) and primary school or below (OR:7.316, 95% CI: 1.619–34.112, *p* = 0.000) were associated with significantly reduced odds of being in the gradual decline group.

The factors predictive of membership in the rapid decline group were distinct. Again, older age was a strong negative predictor (OR: 0.843 per year increase, 95% CI: 0.764–0.929, *p* = 0.001). Similarly, a lower educational level was a major barrier to rapid recovery. Compared to patients with a high school education or above, those with primary school or below had drastically reduced odds of being in the rapid decline group (OR:6.799, 95% CI: 2.895–27.896, *p* = 0.000). Notably, postoperative complications, primary caregiver type, and social support were not statistically significant predictors for this comparison.

**Table 3:** Multivariate logistic regression analysis of factors associated with trajectory group membership (reference: high dependency group)

Variable	Gradual decline group				Rapid decline group			
	OR	CI 95%		*P*-value	OR	CI 95%		*P*-value
LL		UL	LL			UL		
**Age**	0.889	0.838	0.943	0.000	0.843	0.764	0.929	0.001
**Postoperative complications**								
No	7.141	2.029	25.131	0.002	2.298	0.216	24.409	0.490
Yes	1	(Reference)			1	(Reference)		
**Primary caregiver**								
Spouse or child	0.196	0.073	0.525	0.001	0.144	0.020	1.011	0.051
Care attendant	0.710	0.213	2.364	0.576	0.660	0.095	4.565	0.673
None	1	(Reference)			1	(Reference)		
**Education level**								
Primary school and below	7.316	1.619	34.112	0.000	6.799	2.895	27.896	0.000
Junior high school	4.539	1.470	14.013	0.009	2.081	0.107	40.337	0.628
High school and above	1	(Reference)			1	(Reference)		
**Social support**								
Better	0.245	0.084	0.715	0.010	0.277	0.050	1.523	0.140
Poorer	1	(Reference)						

OR: Odds Ratio; LL: Low limit; UL: Upper limit

## Discussion

Our study identified three distinct trajectories of care dependency within 14 days after THA in older patients: high dependency group, gradual decline group, and rapid decline group. Key factors such as age, postoperative complications, primary caregiver, educational level, and social support were significantly associated with these trajectory patterns. In addition, our analysis revealed that the factors distinguishing the high dependency group from the improving groups (gradual decline and rapid decline) followed two distinct patterns. Membership in the gradual decline group was associated with a combination of demographic, clinical, and psychosocial support factors, whereas the transition to the rapid decline group was primarily driven by younger in older patients and higher educational attainment.

Patients in the gradual decline group likely exemplify a compensatory recovery model. They shared significant intrinsic vulnerabilities with the high dependency group, namely older age and a higher burden of postoperative complications, which inherently limit physiological reserve and functional capacity. However, they were buoyed by robust external support systems that enable gradual improvement. The presence of family members as primary caregivers provided indispensable practical assistance for activities of daily living and ensured adherence to rehabilitation protocols, directly mitigating functional limitations [41–43]. Concurrently, strong social support likely operated through psychosocial mechanisms, reducing anxiety and depression, enhancing motivation, and fostering a sense of security, all of which were conducive to recovery [44–46]. The fact that lower education levels remained a negative predictor even in this model suggests that the barriers of lower health literacy were only partially overcome by support, resulting in a slower pace of recovery compared to their more educated counterparts [47, 48].

In contrast, the rapid decline group appeared to be driven by an intrinsic capacity model. It conferred intrinsic resilience and capacity for autonomous recovery. Younger age was arguably the strongest predictor of functional recovery, associated with better physical reserve, neuromuscular control, and healing capacity [49–51]. Higher educational level (with lower education being a strong negative predictor) was a well-established proxy for greater health literacy and socioeconomic advantage [52, 53]. Patients with higher education may more effectively process complex medical information, proactively engage in rehabilitation exercises, communicate their needs to healthcare providers, and problem-solve obstacles during recovery [54–56]. This combination of superior biological capital (younger) and cognitive capital (education) empowers patients to achieve rapid functional gains, often with less dependence on external support systems, which explains why social support and caregiver type were not significant predictors for this group when these core factors are considered.

This dichotomy presented a compelling theoretical framework for post-THA recovery: escape from persistent high dependency can be achieved through one of two pathways: (a) the compensatory pathway, where strong external support counterbalances intrinsic vulnerabilities to facilitate gradual improvement, or (b) the autonomous pathway, where abundant intrinsic resources enable rapid, self-driven recovery. The high dependency group, consequently, represents a population lacking in both.

These findings regarded the negative impact of age and postoperative complications align robustly with the extensive literature on surgical recovery in older adults [57, 58]. The positive role of social support and family caregivers also corroborates previous studies highlighting the importance of the psychosocial environment [59–63].

However, our study significantly extended existing knowledge by demonstrating that these factors do not operate in isolation but form distinct predictive patterns for different recovery velocities. By identifying education as a critical divider between rapid and slow recovery, we move beyond traditional clinical factors and underscore the profound influence of patient empowerment and health literacy, a dimension that has been relatively underexplored in orthopaedic recovery studies.

The identification of heterogeneous trajectories underscored that the post-THA recovery process was not uniform. This finding challenges the traditional “one-size-fits-all” approach to post-operative care and highlights the necessity for early screening and targeted interventions for patients at risk of prolonged high dependency, such as those in the high dependency group.

These findings translate directly into actionable strategies for precision care. For Clinical Practice: We propose a risk-stratification algorithm immediately after admission. Older patients with complications and lower education should be flagged as high-risk for prolonged dependency. For this group, a proactive multidisciplinary approach was essential, including structured pre-operative education, early involvement of social workers to assess and bolster support systems, and planning for intensive home care or transitional care after discharge.

For the high-risk group destined for the compensatory pathway, interventions must fortify their support systems: structured family caregiver education, early discharge planning with social work, and arranged home health services. For those with the capacity for the autonomous pathway, interventions should focus on empowerment: providing advanced self-management resources and setting ambitious mobilization goals.

Future studies should investigate whether interventions aimed at improving health literacy (e.g., through simplified educational materials, teach-back methods, or digital health tools) can mitigate the negative impact of low education and accelerate recovery in high-risk populations.

### Strengths and limitations

Our study has several strengths. First, this was the first study to identify and characterize heterogeneous trajectories of care dependency specifically within the critical two-week post-operative period following THA in older adults. This short-term, dynamic perspective provides novel insights into the early recovery process, which is crucial for timely intervention. Second, the adoption of an analytical approach— Latent Growth Mixture Modeling (LGMM) coupled with multinomial logistic regression—allowed for a more nuanced understanding than traditional methods. LGMM can identified the heterogeneous subgroups with distinct development trajectories hidden beneath the population average, thereby providing a finer-grained perspective for understanding individual differences among patients. This approach not only identifies distinct recovery pathways but also elucidates the differential impact of factors on belonging to one improving group versus another (gradual decline vs. rapid decline), when compared to the high dependency group. Finally, the findings have immediate and actionable clinical implications, providing a evidence-based framework for early risk stratification and the development of targeted support strategies for vulnerable elderly patients.

Notwithstanding these strengths, several limitations of this study should be acknowledged. Firstly, the study sample was recruited from three hospitals in two cities within Hubei Province. Although the sample size was adequate for the statistical analyses performed, the geographical and demographic diversity was limited. It may not be fully representative of the broader national population, limiting the generalizability of our findings. Future multi-center, nationwide studies with larger sample sizes are warranted to confirm our results. Secondly, this study only tracked patients until the 14th postoperative day, which captured only the ultra-short-term recovery phase. These findings were not generalizable to the broader population of THA patients who have shorter, uneventful recoveries. The results should be interpreted as pertaining to a subgroup with high care needs. This short-term focus does not capture the trajectory of care dependence in the mid- to long-term (e.g., 6 months, 1 year, or beyond). Future research with extended follow-up was crucial to understanding the complete recovery process. Thirdly, the primary outcome of care dependence was assessed using patient self-reports, which were susceptible to recall and social desirability bias. Incorporating objective functional assessments in future studies would enhance the robustness of the data. Fourthly, although we included several key covariates, unmeasured confounding factors, such as pre-operative functional status, specific surgical techniques, anesthesia types, and detailed rehabilitation protocols, were not included in our analysis and might influence care dependency trajectories.

## Conclusion

This study not only confirmed the heterogeneity of early care dependency trajectories following THA in older patients but, more importantly, uncovered distinct mechanistic pathways out of a state of high care dependency. The first was a compensatory pathway, where robust external support (e.g., family caregivers and social support) facilitates gradual recovery in patients with intrinsic vulnerabilities. The second was an autonomous pathway, driven primarily by younger in older patients and higher educational attainment, which enables rapid, self-directed recovery. These findings advocated for a paradigm shift from a one-size-fits-all approach to personalized, risk-stratified post-operative care. Early identification of patients at risk for prolonged dependency allows for targeted interventions, tailored to their specific needs, ultimately optimizing recovery outcomes and resource allocation.

## Supplementary Information

Below is the link to the electronic supplementary material.

Supplementary Material 1

## Data Availability

The data are availability from the corresponding author on reasonable request.

## Abbreviations

THA: Total Hip Arthroplasty
LGMM: Latent Growth Mixed Models
WHO: World Health Organization
ADL: Activities of Daily Living
BMI: Body Mass Index
DVT: Deep Vein Thrombosis
CVI: Content Validity Index
