# Supplementary material for: A study on the trajectory of change and influencing factors of care dependence in elderly patients during hospitalization after total hip arthroplasty
Source: BMC Nurs. 2026 Feb 4;25:196. doi: 10.1186/s12912-026-04383-8 (PMC12958670; doi:10.1186/s12912-026-04383-8)
Supplement: Supplementary file 1 — Supplementary Material 1 [file 12912_2026_4383_MOESM1_ESM.pdf]

Appendix I Informed Consent Form

Dear Patient and Family Members,

Hello! We are researchers from Yangtze University. We are currently conducting a research project on postoperative care dependency in elderly patients who have undergone total hip arthroplasty. We would like to invite you to participate in this research and appreciate your support and cooperation.

This study primarily involves a questionnaire survey. You may choose whether to participate, and you may withdraw at any time during the process. We fully respect your right to decline and will honor your decision. To protect your privacy, all information obtained through the questionnaire will be kept strictly confidential. The survey results and all collected data will be used solely for this research project and will be anonymized during processing. All survey participants and data compilers will strictly adhere to confidentiality agreements.

Completing this questionnaire will take approximately 30 minutes. When answering, please select the responses that most accurately reflect your genuine feelings without reservation. Please ensure all questions are answered. Thank you.

If you fully understand the above and agree to participate in this interview, please sign your name in the space below. Your signature indicates your permission for our research and does not affect your ability to protect your legal rights and interests. This study has been approved by the Ethics Committees of the First Affiliated Hospital of Yangtze University, Yichang Central People's Hospital, and Yichang First People's Hospital. Thank you very much for your support!

Informed Consent Statement:

☐ I have been informed of the purpose, background, procedures, potential risks, and benefits of this study. I have had sufficient time and opportunity to ask questions, and I am satisfied with the answers provided.

☐ I have also been informed of whom to contact if I have questions, wish to report difficulties or concerns, have suggestions regarding the study, or wish to obtain further information or provide assistance for the research.

☐ I understand that I may choose not to participate in this study, or I may withdraw at any time during the study without providing any reason, and this will not prejudice any of my entitlements.

☐ I will receive a copy of this informed consent form, containing both my signature and the researcher's signature.

☐ I have read this informed consent form and agree to participate in this study.

Participant Signature: ________ Date: ________

Participant Contact Information_______________

☐ This informed consent form is signed by the guardian on behalf of the participant due to the participant's lack of capacity or limited capacity.

Guardian Signature: ________ Date: ________

Guardian Contact Information: _____________

Researcher's Declaration:

☐ I confirm that I have explained and discussed the nature, purpose, requirements, and potential risks of this study with the participant, along with discussing other available treatment options. I also confirm that a copy of this informed consent form has been provided to the participant for their records. The participant has understood and agreed to participate in this study.

Researcher Signature: ________ Date: ________

Appendix II General Information Questionnaire

Please mark " √ " in the "◯" before the option that matches your true situation, or fill in the appropriate text in the " ".

1. Name:

2. Hospital ID Number:

3. Gender [Single Choice]*

◯ Male

◯ Female

4. Age:

5. Marital Status [Single Choice]*

◯ Married

◯ Unmarried (including never married, divorced, widowed)

6. Educational Attainment [Single Choice]

◯ None

◯ Primary School

◯ Junior High School

◯ High School

◯ College or Higher

7. Primary Caregiver [Single Choice]*

◯ Spouse

◯ Children

◯ Caregiver

◯ None

8. Living Arrangement [Single Choice]*

◯ Living Alone

◯ Living with Spouse

◯ Living with Child

◯ Living in Nursing Home

◯ Living with Spouse and Children

9. Medical Expense Payment Method [Single Choice]*

Out-of-Pocket

◯ Urban Employee Basic Medical Insurance

◯ Urban Resident Basic Medical Insurance

◯ New Rural Cooperative Medical Insurance

◯ Commercial Insurance

10. Monthly Per Capita Household Income [Single Choice]*

○ <3000

○ 3000≤Y<5000

○ 5000≤Y<8000

○ ≤8000

(Y: Income)

11. Occupation [Single Choice]*

○ Retired

○ Worker

○ Farmer

12. Surgical Anesthesia Method:

13. Presence of Other Diseases [Single Choice]*

○ Yes ________________*

○ No

14. BMI Nutritional Score:

15. Activities of Daily Living (ADL) Score:

16. Pain Score:

17. Deep Vein Thrombosis Risk Score:

18. Pressure Injury Score:

19. Postoperative Use of Analgesic Pump [Single Choice]*

○ Yes

○ No

20. Geriatric Depression Scale Score:

21. Time to first ambulation:

22. Time to first oral intake:

Appendix III Chinese Version of the Care Dependency Scale

This section measures your current level of care dependency. Please select the option that best matches your current situation and feelings, and mark "√" on the corresponding option.

Item No.	Item Name	Specific Description	Completely Dependent	Mostly Dependent	Partially Dependent	Slightly Dependent	Almost Independent
1	Eating	Ability to feed oneself (eat solid food and drink liquids) without assistance.					
2	Elimination	Ability to independently control and manage urination and defecation.					
3	Posture Maintenance	Ability to independently assume and maintain a comfortable posture (e.g., lying, sitting) without support.					
4	Mobility	Ability to independently get out of bed, walk, and move around (including using					

		routine assistive devices like walkers).
5	Circadian Rhythm	Ability to maintain a normal sleep-wake cycle (no insomnia at night, no excessive sleepiness during the day, no reversal of day and night).
6	Dressing/Undressing	Ability to independently put on/take off clothing, shoes, and fasteners (e.g., buttons, zippers).
7	Body Temperature Regulation	Ability to independently adjust clothing or use aids (e.g., blankets) to maintain a comfortable body temperature in response to external weather changes.
8	Personal Hygiene	Ability to independently perform personal cleanliness tasks (e.g., face-washing, toothbrushing, hair grooming, basic bathing).
9	Risk Avoidance	Ability to recognize and avoid potential hazards (e.g., falling out of bed, slipping while walking) to ensure personal safety.
10	Communication	Ability to independently express needs, thoughts, or feelings to others (verbally or via alternative communication tools like picture cards).
11	Social Contact	Ability to independently maintain regular contact with family or friends (e.g., making phone calls, meeting in person) without prompting.
12	Adherence to Rules & Values	Ability to independently understand and follow basic rules (e.g., hospital visiting hours) or personal ethical principles.
13	Daily Activity Planning	Ability to independently organize routine daily activities (e.g., arranging meal times, rest periods, or light exercises).
14	Social/Recreational F	Ability to independently participate in social or recreational activities outside the home (e.g., community events, hobby groups) when physically capable.
15	Learning Ability	Ability to independently acquire new skills (e.g., using a new assistive device) or retain previously learned skills without additional teaching.

Appendix IV Chinese Version of the Groningen Orthopedic Social Support Scale (GO-SSS)

This section measures your current level of social support. Please select the answer that best reflects your current situation and feelings, and mark "√" on the corresponding option.

Item No.	Statements About Social Support	Never	Occasionally	Frequently	Regularly
1	My family and/or friends understand me.				
2	My family and/or friends assist me with exercises.				
3	My family and/or friends prepare meals for me.				
4	My family and/or friends listen to me when I want to talk.				
5	My family and/or friends stay with me when I am ill.				
6	I can share the problems that trouble me with my family and/or friends.				
7	My family and/or friends help me buy things I need.				
8	My family and/or friends are present in a timely manner when I need them.				
9	My family and/or friends help me go out (e.g., for medical appointments, errands).				
10	I can share my joys and sorrows with my family and/or friends.				
11	My family and/or friends help me with housework.				
12	My family and/or friends help me make decisions when I need it.				
